# Determinants and Prevalence of Tobacco Smoking among Medical Students at Jazan University, Saudi Arabia

**DOI:** 10.1155/2021/6632379

**Published:** 2021-02-03

**Authors:** Mohammed Alkhalaf, Abdullatif Suwyadi, Eissa AlShamakhi, Hassan Oribi, Zain Theyab, Ibrahim Sumayli, Abuobaida Yassin, Abdulwahab Aqeeli, Ahmad Alqassim

**Affiliations:** ^1^Faculty of Medicine, Jazan University, Jazan, Saudi Arabia; ^2^Internal Medicine Department, Faculty of Medicine, Jazan University, Jazan, Saudi Arabia; ^3^Family and Community Medicine Department, Faculty of Medicine, Jazan University, Jazan, Saudi Arabia

## Abstract

Tobacco smoking has a significant role in health deterioration worldwide; it can lead to many dangerous diseases. Tobacco smoking among medical students is common worldwide, but the prevalence and determinants vary from one community to another. Data from medical students in Saudi Arabia is limited. This study was conducted to estimate the prevalence and determinants of smoking among medical students at the College of Medicine, Jazan University, Saudi Arabia. A cross-sectional study using a self-administered electronic survey was conducted to estimate tobacco smoking's prevalence and characteristics among medical students at Jazan University. The survey includes information on the gender, academic year, academic performance, type of tobacco smoking, and age of onset of the participants' tobacco smoking. Other data, like the prevalence of passive smoking and social factors, were considered, too. The sample size was 354, 51.7% males and 48.3% females, students with a response rate of 38.02%. The prevalence of smoking among medical students was 12.4%, while passive smoking prevalence was 39.9% of all medical students. The research shows that 18.6% of male and 5.9% of female medical students were active smokers. Regarding the type of tobacco, we found that 47% of male smokers used waterpipe, while the percentage among female smokers using waterpipe reached 77.8%. The age of onset of smoking for 34.9% of the smokers was between 18 and 21 years old. The prevalence of smoking is inversely proportional to the GPA. Additionally, 71.1% of the smokers did not have a smoker friend, and only 13.3% of the smokers were motivated to quit. University age is critical for smoking habits, and the smoking cessation rate was low. More campaigns should be done in universities to increase smoking cessation awareness, and smoking cessation clinics should be activated at universities.

## 1. Introduction

Smoking is a significant health hazard. It contributes to morbidity and mortality of many diseases in the heart and lungs [1, 2]. Tobacco smoking is the leading cause of cancer of the lung, upper respiratory tract, gastrointestinal tract, kidneys, pancreas, liver, bladder, and cervix and leukemia [3]. Tobacco smoking is known to cause heart disease, stroke, and chronic obstructive pulmonary disease (COPD), which includes emphysema and chronic bronchitis [4]. Smoking also increases the risk for tuberculosis, certain eye diseases, and the immune system's problems, including rheumatoid arthritis. Smoking costs nearly 6% of the total global healthcare expenditure and almost 2% of the global gross domestic product. In 2012, smoking-related illness accounted for 12% (2.1 million) of all deaths among working-age adults aged 30–69 years, with the highest proportion in Europe and America [5].

Tobacco smoking among students is hugely widespread; 47.5% of students occasionally observed symptoms like cough attributed to smoking [6]. Most long-term smokers start smoking from 18 to 25 years of age [7]. It is believed that young adults show their power and importance through smoking; some smoke to join their social group and build their social network or relieve stress [8]. Smoking initiation in adolescence increases the likelihood of continued smoking and reduces discontinuation [9]. The transition from high school to college is critical for adopting a healthy lifestyle and habits [10]. Although 41% of smokers annually try to stop smoking, relapse is common, and abstinence is achieved by only 10% [11].

In a study conducted in 2010 in Riyadh, Saudi Arabia, 14.5% of university students smoked, with a prevalence among male students of 32.7% and 5.9% in females [12]. In that study, 51% of males smoked cigarettes, and 37% smoked waterpipes, while 35% of females smoked cigarettes and 33% smoked waterpipes and 32% did not indicate those details [12]. In 2013, the prevalence of tobacco smoking among intermediate and secondary school students in the Jazan region on both male and female was 10.7%. The prevalence of tobacco smoking was higher among students who had poor academic performance [13]. In another study in 2014 conducted in Jazan with participants from three areas of the Jazan region (Jazan University, Technical College, and Academic Health College), the prevalence of tobacco smoking was 16.8%, male smokers were 25.6%, and female smokers were 4.6% [14]. In a 2005 study at King Saud University, Riyadh, Kingdom of Saudi Arabia, 3% of male medical students were currently active smokers, 5.3% were ex-smokers, and 38% were passive smokers. The most common reason for smoking behavior was friends' influence (35.6%) [15].

Although multiple studies have been conducted in Saudi Arabia regarding tobacco smoking, relatively few researches have highlighted the medical students' smoking behavioral patterns. There was an inconsistency regarding the prevalence of tobacco smoking among medical students. Medical students have high levels of stress than other undergraduate students, and stress is a significant reason young people take up smoking [16]. Some other studies found that tobacco smoking prevalence was less among medical than nonmedical students [17]. This study was conducted to identify the prevalence of smoking among medical students at Jazan University, Saudi Arabia, to develop the best control measures related to smoking consumption. We also looked to determine the prevalence of tobacco smoking among male and female medical students, used tobacco form, passive smoking status, academic performance among smokers, and other related factors.

## 2. Materials and Methods

### 2.1. Study Area, Design, and Population

This study was conducted in the Faculty of Medicine at Jazan University in Saudi Arabia, with approximately 931 students, including 453 males and 478 females. Jazan (also called the Gizan) region is one of the thirteen areas of Saudi Arabia. Jazan is located on the southwestern side of the country and populated with more than 1.5 million residents. Our study design was an observational, descriptive cross-sectional, self-administered electronic survey targeted at Jazan University medical students registered for the academic year 2019/2020.

### 2.2. Sampling Procedures

The sample size was determined based on the estimated prevalence of tobacco smoking in Jazan, Saudi Arabia, 16.8%, a confidence interval of 95%, error not exceeding 5%, and a nonresponse rate of 50%. Using these parameters, a total of 350 students were estimated as the study sample size. The sampling frame was prepared in consultation with the admission office at the College of Medicine, and details of the number of medical students were obtained.

The research team contacted the group leader of each academic year in the college to reach students and requested them to share the electronic survey link to all students in their respective academic year. To ensure receiving a valid number of responses, the electronic link was shared with all medical students at the college.

### 2.3. Data Collection Instrument

Data was collected using an Arabic self-administered structured questionnaire. The questionnaire was adapted from the Global Adult Tobacco Survey questionnaire (GATS) [18]. The questionnaire included demographic data (age, sex, year of study, and academic performance), tobacco smoking, tobacco use pattern (the type of smoking and age of initiation), and passive smoking. A smoker was defined as a student who currently used at least one tobacco product. A passive smoker was described as a student who has a family member or a close friend who is a smoker.

### 2.4. Data Management, Analysis, and Software

The data were analyzed using Stata/ICTM 13.1 (StataCorp LP). Data analysis involved descriptive and inferential statistics. Gender, year of the study, and GPA were reported using descriptive statistics.

Chi-square or Fisher's exact test was conducted to examine the relationships between different demographic data and smoking status. Fisher's exact test was used to test gender differences in the type of smoking and age of onset of smoking. *α* = 0.05 value or less was used for determining statistical significance.

### 2.5. Ethical Considerations

Ethical clearance was approved by the Medical Research Ethical Committee, Faculty of Medicine, Jazan University, with reference number (REC40/3-075). The individual consent from each student to participate in the study was a prerequisite for data collection. On the front page of the survey, a statement declared that answering the survey meant the agreement for participating in the study. All information was kept confidential and was not accessed except for scientific research purposes.

## 3. Results

A total of 354 medical students completed the study questionnaires, yielding a response rate of 38% (*n* = 354/931). As shown in Table 1, male medical students were 183 (51.7%) and female medical students were 171 (48.3%) of the total sample. The academic year of the participants ranged from 1st to 5th year. The results showed that 44 (12.4%) of the medical students were smokers, 34 (18.6%) of male medical students were smokers, while 10 (5.9%) of female medical students were smokers. According to the year, the highest rate of smokers was in the 3rd year, in which 14 (15.1%) of them were smokers, and the lowest rate was in the 2nd year, in which 9 (8.6%) of them were smokers. Moreover, most of the medical student's GPA was between 4.49 and 3.75, and this represents 40.7% of the total sample with no significant difference between smokers and nonsmokers (*p* = 0.011).

Regarding smoking, the highest standard of smoking was waterpipe with 23 students (53.5%). According to the gender, the top type of tobacco for male and female smokers was waterpipe, with 15 male students (42.85%) and 8 (80%) female students, as seen in Table 2.

According to the age of onset of smoking, the results showed that from the total sample of medical students, 15 (34.9%) of smokers started smoking between 21 and 18 years old, and for the male smokers, 12 (80%) of them started smoking between 21 and 18 years old. Female smokers and 3 (20%) started smoking between 21 and 18 years old (Table 2).

Table 3 shows that 141 (39.9%) of medical students were passive smokers.  Table 4 shows that 13 (28.9%) of the medical student smokers had a smoker friend. Only 6 (13.3%) of smoking medical students were motivated to quit smoking (Table 5).

## 4. Discussion

This study was conducted to assess the prevalence of smoking among medical students at Jazan University, Saudi Arabia. The prevalence of smoking in our study was 12.4% [12, 15]. The similarity in the smoking prevalence among students is expected since they will be exposed to the same stress levels [19, 20]. Previous studies in Jazan reported a smoking prevalence range of 10.7-16.8%, consistent with our findings [13, 14].

Male smokers in our study represented 18.2%, while females were only 5.9% (Table 1). A study has reported the highest prevalence of smoking among males in Saudi Arabia (42.3%) [21]. On the other hand, one study reported the lowest prevalence among males of 15.6% [22]. Among studies reporting female smoking, the prevalence range was 10.3% and 0.9% [23, 24]. The significant difference between males and females in Saudi Arabia may be attributed to the limited access to female participants and social desirability bias tied to smoking behavior among females [25].

The prevalence of smoking in the first year (15.9%) was the highest, and this can be explained by the stress in the new college environment, as mentioned in previous studies [26, 27]. Except for the third year (15.1%), the prevalence of tobacco smoking was comparable. The third year in medical school is the transition between the basic and clinical years, and this could increase the stress, which contributes to the increased smoking rate among students.

According to the students' academic performance, tobacco smoking prevalence was inversely proportional to the GPA (Table 1). One-third of the students with a GPA of less than 2.75 were smokers, while only 9% of the students with a GPA of 4.50–5.00 were smokers. Hence, the distribution of smokers' GPA in this study agrees with that found in intermediate and secondary schools at Jazan. Findings show that the most independent predictor of smoking was academic performance [13].

Among smokers in our study, the majority of students used smoke waterpipe (53.5%), cigarette (25.6%), and both waterpipe and cigarette (9.3%), as shown in Table 2. These results are comparable to a previous study conducted among Taibah University students in Medina, Saudi Arabia [28]. Based on gender specificity, the Jazan male and female medical students who smoke using waterpipe or cigarette were 76.5% and 88.9%, respectively. In Medina, the authors found that 62.3% of males used waterpipes or cigarettes, while 58.8% of females did so [28]. These results indicate that the majority of young smokers prefer cigarettes and waterpipes as tobacco smoking tools. Such information could be emphasized during future health education campaigns. The differences between the studies are attributed to the different study populations.

In this study, the age of onset of smoking according to smokers was between 21 and 18 years old (55.6%), which agrees with earlier studies which stated that the transition from high school to college is a critical period for smoking, and the age between 21 and 18 years old is when most of the students transfer to college as shown in Table 2 [10]. Besides, the vast majority of long-term smokers start smoking between 10 and 25 years, which means that university students who start smoking may persist in smoking for an extended period. The prevalence of passive smoking (second-hand smoking) in this study (39.9%) was similar to Al-Turki (38%) [15].

Many studies talk about friends' influence on smoking behavior; as mentioned, students who have smoker friends are five times more likely to smoke. The most common reason for smoking behavior in another study was friends' influence (35.6%) [12, 29]. In this study, 28.9% of smokers had a smoker friend. Furthermore, previous research shows that 41% of smokers annually try to stop smoking [11]. In our study, only 13.3% of the smokers were motivated to quit smoking, which unfortunately represents a low rate.

In our study, we tried to collect a larger sample. However, the university was suddenly closed due to the coronavirus pandemic, and all students transitioned to online studying, which made it more difficult for us to reach them. However, we were able to recruit 38% of the whole study population.

## 5. Conclusions

The prevalence of smoking among medical students at Jazan University, Saudi Arabia, is 12.4%. According to the results obtained, 18.6% of male medical students and 5.9% of female medical students are smokers. Since the university age is critical for establishing the smoking habit, which has so many dire consequences, this study's prevalence should be paid attention to. Health education campaigns should be conducted in universities to increase students' awareness regarding this topic and explain the long-term risks of smoking. Additionally, it would be beneficial to establish smoking cessation clinics on campuses.

## Figures and Tables

**Table 1:** Comparison of students' demographics according to smoking status.

Characteristics	Smoker	Nonsmoker	Total	*p* value (chi-square test)
Total sample	44 (12.4%)	310 (87.6%)	354	
Gender				
Male	34 (18.6%)	149 (81.4%)	183	<0.001
Female	10 (5.9%)	161 (94.1%)	171	
Year of study				
1	10 (15.9%)	53 (84.1%)	63	0.6
2	9 (8.6%)	96 (91.4%)	105	
3	14 (15.1%)	79 (84.9%)	93	
4	6 (11.5%)	46 (88.5%)	52	
5	5 (12.2%)	36 (87.8%)	41	
GPA out of 5.00				
Less than 2.00	1 (100%)	0 (0%)	1	0.011^∗^
2.00 to 2.74	7 (33.3%)	14 (66.7%)	21	
2.75–3.74	11 (11.1%)	88 (88.9%)	99	
3.75–4.49	17 (11.8%)	127 (88.2%)	144	
4.50–5.00	8 (9%)	81 (91%)	89	

^∗^Fisher's exact test.

**Table 2:** Type of smoking and age of onset of smoking among tobacco smokers by gender.

	Male (*n* = 34)	Female (*n* = 9)	Total (*n* = 43)	*p* value (Fisher's exact test)
Type of smoking				
Cigarette	10 (90.9%)	1 (9.1%)	11	0.465
Waterpipe	16 (69.6%)	7 (40.4%)	23	
Both	4 (100%)	0 (0%)	4	
Other (cigars and e-cig^∗^)	4 (80%)	1 (20%)	5	
Age of onset of smoking				
Less than 10	2 (40%)	3 (60%)	5	
10-13	6 (85.7%)	1 (14.3%)	7	0.282
14-17	10 (90.9%)	1 (9.1%)	11	
18-21	12 (80%)	3 (20%)	15	
22 or above	4 (80%)	1 (20%)	5	

^∗^e-cig: electronic cigarettes.

**Table 3:** Prevalence of passive smoking of the total sample.

Passive smoking	Frequency
Yes	141 (39.9%)
No	213 (60.1%)

**Table 4:** Smokers who had a smoker friend.

Had a smoker friend	Frequency
Yes	13 (28.9%)
No	32 (71.1%)

**Table 5:** Smokers who were motivated to quit smoking.

Motivated to quit	Frequency
Yes	6 (13.3%)
No	39 (86.7%)

## Data Availability

Data are available on request.
